# Early Experience with Biobranetm for Definitive Coverage of Tangentially Excised Partial-Thickness Thermal Burns

**DOI:** 10.29252/wjps.10.1.119

**Published:** 2021-01

**Authors:** Christopher Wei Guang Ho, Jia Le See, Shi-Hui Yang, Bien Keem Tan

**Affiliations:** 1 Department of Plastic, Reconstructive and Aesthetic Surgery, Singapore General Hospital, Singapore;; 2 Department of General Surgery, Singapore General Hospital, Singapore

**Keywords:** Biobrane, Burns excision, Partial-thickness burns, Skin substitute, Wound healing

## Abstract

Biobrane^TM ^is a popular biosynthetic semi-permeable skin substitute conventionally applied onto non-excised partial thickness burn wounds to facilitate healing. The use of Biobrane^TM^ for definitive coverage after excision of partial-thickness thermal burns has not been reported. We highlighted our experience of immediate Biobrane^TM^ application for definitive coverage of tangentially-excised partial thickness thermal burn wounds in four patients. This technique is safe and efficient, minimizes painful and costly dressing changes, avoids the complications associated with autologous skin grafting, and eliminates the unpredictability of burns wound conversion. We believe this method expands the indications for Biobrane^TM^ usage, accelerates wound healing, and provides better aesthetic outcomes.

## INTRODUCTION

Early excision and autologous skin grafting is the gold standard treatment for burns.^1^ However, skin grafting is not without significant morbidity, including donor site bleeding, infection, dysaesthesia, abnormal scarring and dyschromia. The cosmetic appearance of skin grafted sites is also frequently unsatisfactory.^2^ Introduced in 1979, Biobrane^TM ^(UDL Laboratories, Rockford, IL) is a highly-conformable synthetic acellular material comprising of a flexible inner trifilament nylon fabric mesh embedded with hydrophilic type I porcine collage and an outer semipermeable silicone membrane.^3^^,^^4^

When placed on a clean partial-thickness burn, collagen on the inner surface preferentially binds to fibrin from the wound exudate. Adherence of Biobrane^TM^ to the wound reduces pain and permits early joint mobilization. The silicone membrane functions as a protective epidermal barrier against infection and prevents wound desiccation, whilst permitting water vapour permeability to prevent fluid from collecting.^3^^,^^4^

Over the next 10-14 days, proliferating epithelial cells from hair follicles within the deep dermis migrate across the wound surface, forming ever-enlarging epidermal islands that eventually become confluent, and in the process causing spontaneous detachment of Biobrane^TM^ from the re-epithelised wound.^3^^,^^4^

During this period, only the gauze dressings on top of the Biobrane^TM^ is changed, which reduces patient discomfort and duration of hospitalization. As Biobrane^TM^ is transparent, tracking the progress of wound healing and monitoring for potential complications is straightforward.^3^^,^^4^ Numerous authors have shared their protocols and techniques to maximise successful use of Biobrane^TM^.^4^^-^^6^ Wound selection is paramount: an exudative wound bed is a prerequisite for adherence, hence Biobrane^TM^ should not be applied to non-exudative deep dermal and full-thickness burns that require excision and skin grafting. Meticulous aseptic wound bed cleaning, as well as removal of unviable blisters and surface debris is also essential to prevent Biobrane^TM^ infection and premature separation from the wound bed.^5^

Biobrane^TM^ should be applied within 24-48 hours after the burn injury, beyond which the risks of wound colonization leading to prosthesis infection and failure become significant.^4^^-^^6^ Biobrane^TM^ is primarily utilised as a dressing for non-excised superficial and mid-dermal thermal burns, as well as for skin graft donor sites, to expedite re-epithelization. Biobrane^TM^ has also been deployed for temporary coverage of excised deep dermal and full-thickness thermal burns to prepare the wound for autografting,^7^ and has reportedly been used for definitive coverage of partial-thickness alkaline burns with good results.^8^ The use of Biobrane^TM ^for immediate definitive coverage of thermal burn wounds that have been tangentially excised has not been described in the literature. We reported our experience using this method on four patients with partial-thickness thermal burns. 

## CASE REPORT

Written informed consent was obtained from each patient. After induction of general anaesthesia and aseptic cleansing, the area of burns to be excised received a subcutaneous injection of tumescent solution (adrenaline 1:250,000, gentamicin 80 mg and lignocaine 3 mg/kg in 500 mL of Ringer’s lactate). A tourniquet was not utilized. Ultra-thin tangential excision was performed with an air-powered dermatome (Zimmer Biomet, Warsaw, IN) at 4/1000 inch cutting thickness until uniform punctate bleeding was observed from the wound. Topical adrenaline solution-soaked laparotomy sponges were then applied for hemostasis. Biobrane^TM^ was secured to the wound under slight tension with sterile adhesive tape (Hypafix; Smith&Nephew, Memphis, TN) and surgical staples, and wrapped in gauze soaked with povidone-iodine (Betadine; Pfizer, West Ryde, Australia). 

***CASE 1***

**Fig. 1:**
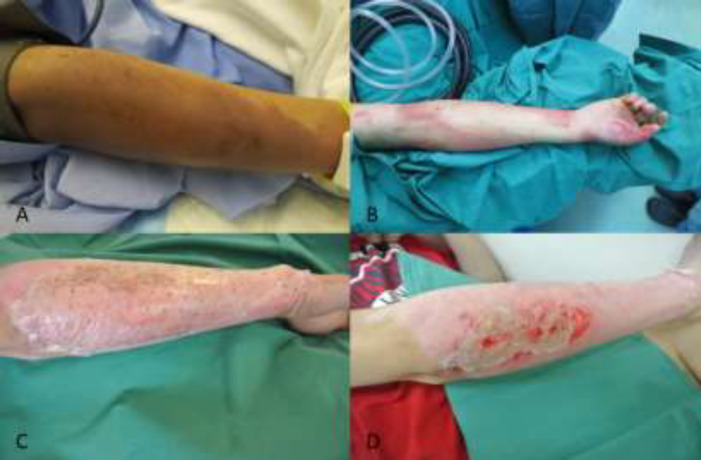
**Case 1:** **(A):** Right forearm burns on presentation. **(B):** The lateral aspect (1% TBSA) was poorly blanching after de-blistering and subsequently tangentially excized. Biobrane^TM^ was applied to all burns (excised and non-excised). **(C):** All wounds were healing well on POD 10. **(D):** Epithelization was complete by POD 15

A 27-year-old male construction worker sustained 15.5% total body surface area (TBSA) partial-thickness thermal burns from a flash fire at an industrial site (Figure 1A). The time interval from accident to surgery was 48 hours. After removal of blisters and aseptic cleansing, the lateral aspect of his right forearm (approximately 1% TBSA) was noted to have sluggish capillary refill (Figure 1B). This area was tangentially excised until punctate bleeding was seen. Biobrane^TM^ was then applied to all burns. The area of excised burns was observed to be healing well underneath the Biobrane^TM^ dressings (Figure 1C) on post-operative day (POD) 10, and had fully epithelized by POD 15 (Figure 1D). 

***CASE 2***

**Fig. 2:**
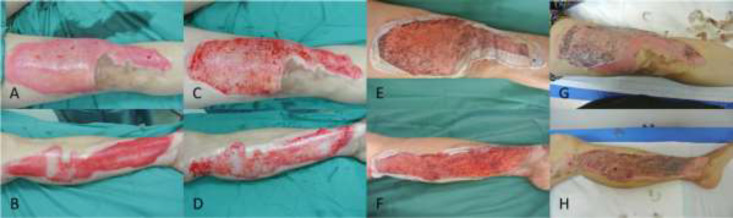
**Case 2. (A and B):** Right thigh and upper shin (6% TBSA) and left calf (4% TBSA) burns with sluggish capillary refill and areas of fixed-staining after debridement. **(C and D):** Tangential excision was performed, and Biobrane^TM^ was applied to the wounds. **(E and F): **The wounds were healing well on POD 7. **(G and H):** Epithelization was complete by POD 11

A 57-year-old female waitress presented six days after sustaining 10% TBSA partial-thickness thermal burns from being scalded by boiling soup at work. After debridement of blisters, her right thigh and upper shin (6% TBSA) and left calf (4% TBSA) were found to be poorly blanching with multiple areas of fixed-staining (Figure 2A and B). These areas were tangentially excised until punctate bleeding was seen from the dermis of the wound (Figure 2C and D). Biobrane^TM^ was then applied to these excised areas. Her wounds were observed to be healing well on POD 7 (Figure 2E and F), and epithelization was complete by POD 11 (Figure 2G and H). A summary of the 4 cases is provided in Table 1. 

The study was performed in accordance with the principles of the Declaration of Helsinki. Written informed consents were obtained. Institutional review board/ethics committee approval was not required for retrospective case reports. Patients provided written informed consent for the publication and the use of their images. Written informed consent was obtained from the patient for publication of this case series and accompanying images. A copy of the written consents was available for review by the Editor-in-Chief of this journal on request.

**Table 1:** Patient demographics and outcomes

**Patient no.**	**Gender**	**Age** **(years)**	**TBSA burns (%)**	**TBSA excised (%)**	**Excision site**	**Time from burns to excision and Biobrane** ^TM^ ** (hours)**	**Time from excision to re-epithelisation (days)**
1	Male	27	15.5	1	Right forearm	48	7
2	Female	57	10	10	Right thigh and shin, left calf	168	11
3	Male	56	18	5	Left arm and forearm	72	8
4	Female	64	14	9	Bilateral thighs	96	13


## DISCUSSION

Contemporary guidelines on Biobrane^TM^ usage recommend that to be applied to newly-burnt, exudative, partial-thickness wounds that have been carefully prepared.^4^^-^^6^ This maximises adherence and reduces the risks of wound colonization and consequent prosthetic infection and failure that plagued many earlier studies.^9^^,^^10^ Our case series demonstrated that ultra-thin, tangential excision allowed conversion of an older (>48 hours) burn to a fresh burn which permitted successful application of Biobrane^TM^. Not only does this remove colonizing bacteria, the raw excised surface also produces renewed exudate, enhancing the adherence of Biobrane^TM^ to the burn wound.^5^

As illustrated above, this technique lends itself well to burns of homogenous or heterogenous depth. If capillary refill is uniformly reduced across the burn, the entire wound is tangentially shaved and Biobrane^TM^ is applied. If a part of the wound is poorly-blanching, only that particular area requires tangential excision, and Biobrane^TM^ can then be applied to the entire wound (excised as well as non-excised). The latter extends the “trial-of-life” concept, where Biobrane^TM ^is applied over burns of uncertain dermal depth in a wound that is hopefully still capable of spontaneous healing.^5^

Numerous groups,^11^^,^^12^ including our own^13^ have shown that Biobrane^TM^ has reduced the need for autologous skin grafting in partial-thickness burns. By improving adherence of Biobrane^TM ^to areas of deeper injury, this novel technique potentially decreases the need for autologous skin grafting (and its attendant cosmetic and functional complications) even further. Nonetheless, it is imperative that patients should be counselled pre-operatively that Biobrane^TM ^may not adhere or “take” over the excised areas and that subsequent re-excision with autologous skin grafting may be necessary. 

Caution should be exercised when applying this technique to deep dermal and full-thickness burns, in these wounds it is highly unlikely that the remnant dermis will be able to produce enough exudate to allow sufficient Biobrane^TM^ adherence after tangential excision. Laser Doppler imaging may be a useful adjunct in determining burn depth (and the suitability of this technique), if uncertainty persists after clinical assessment.^14^ Greenwood *et al.* have warned about the risk of applying Biobrane^TM^ to an excised wound, with blood clots and subsequent fibrovascular ingrowth into the nylon scaffold making removal difficult.^5^. 

We thus paid meticulous attentiontowards achieving hemostasis using a combination of adrenaline-containing tumescent infiltration, bipolar electrocautery and manual pressure. Biobrane^TM ^was only secured onto the raw wound surface, once all visible bleeding had been arrested. We did not encounter any problems related to spontaneous detachment of Biobrane^TM ^from the wound, once epithelization had occurred. It has been demonstrated that Biobrane^TM^ is capable of controlling bacterial growth in wounds with an initial bacterial count of less than 10^5 ^per gram of tissue, and wounds with a bacterial count exceeding 10^5 ^per gram of tissue precludes Biobrane^TM ^“take”.^4^^,^^6^^,^^15^

As burn wounds become progressively colonized by bacteria with time, most authors advise that Biobrane^TM ^is applied within 48 hours of injury,^4^^-^^6^ with a recent retrospective cohort study showing evidence of significantly faster re-epithelization and decreased need for skin grafting, if Biobrane^TM^ is applied within 12 hours.^12^ Nonetheless, there is no conclusive evidence that its use is contraindicated in older burns.^4^^,^^6^ We successfully performed ultra-thin tangential excision with immediate Biobrane^TM^ coverage on burns with a mean age of 105.5 hours (4.4 days; range: 48-168 hours) from the time of injury without any infectious complications. Nonetheless, we do not recommend that this technique is attempted on burn wounds that display overt clinical signs of infection. 

This approach allows more definitive intervention on burns of equivocal healing potential, the excized surface exudes fibrin that promotes binding to collagen on the inner surface of Biobrane^TM^. This promotes greater adherence of Biobrane^TM ^to the wound, and in so doing facilitates accelerated re-epithelisation.^5^^,^^6^ The correlation between burns healing time and subsequent development of hypertrophic scarring and contractures has been widely reported, with burns taking more than 21 days to heal at significantly greater risk of hypertrophic scarring and its sequelae (e.g. unsatisfactory cosmesis, pain, itching, and joint contractures).^2^

More recent work has shown that the rates of hypertrophic scar are multiplied by 1.138 for every additional day taken for the burns to heal (even within the 21 day period); therefore, every effort should be made to accelerate wound healing.^16^ In our study, all excised wounds re-epithelised after an average of 9.75 days (range 7-13 days), and none had developed hypertrophic scarring at 12 months of follow-up. Biobrane^TM^ was shown to be a versatile alternative to conventional dressings in the management of thermal burns. While its cost, benefit ratio has yet to be fully elucidated; Biobrane^TM^ permits reduced frequency and discomfort of dressing changes, potentially reduces the extent of and the need for skin grafting, as well as decreases hospitalization time.^5^^,^^6^^,^^17^^-^^19^

However, current recommendations may restrict its usage. Although the small population and descriptive nature of this series make it impossible to perform any meaningful statistical analysis, these promising early results challenge the widely-accepted doctrine that application of Biobrane^TM^ should be limited to superficial to mid-dermal partial-thickness thermal burns within 48 hours of injury. Preparatory tangential excision expands the indications for Biobrane^TM^ use, allowing earlier definitive treatment of equivocal burns, whilst simultaneously avoiding the complications of skin grafting and retaining the benefits of Biobrane^TM^ that have made it so popular. Larger prospective case-controlled studies are required to further establish the average time to wound healing, cost-effectiveness, outcomes (functional and aesthetic) and complications of this novel technique.

## CONCLUSION

Ultra-thin tangential excision of partial-thickness thermal burns with equivocal capacity for spontaneous healing followed by immediate definitive coverage with the biosynthetic dressing Biobrane^TM^ can achieve good functional and cosmetic results. Contrary to existing guidelines, this technique can be successfully employed on burns that are more than 48 hours from the time of injury. This accelerates re-epithelisation, minimizes the complications of delayed wound healing and may reduce the need for patients to undergo autologous skin grafting. 
